# HoLLiECares - Development of a multi-functional robot for professional care

**DOI:** 10.3389/frobt.2024.1325143

**Published:** 2024-10-09

**Authors:** Julian Schneider, Matthias Brünett, Anne Gebert, Kevin Gisa, Andreas Hermann, Christian Lengenfelder, Arne Roennau, Svea Schuh, Lea Steffen

**Affiliations:** ^1^ Institute of Control Systems (IRS), Karlsruhe Institute of Technology (KIT), Karlsruhe, Germany; ^2^ German Institute of Applied Nursing Research (DIP), Cologne, Germany; ^3^ August-Wilhelm Scheer Institute for digital products and processes gGmbH, Saarbrücken, Germany; ^4^ ArtiMinds Robotics GmbH, Advanced Robotics Section, Karlsruhe, Germany; ^5^ Fraunhofer Institute of Optronics, System Technologies and Image Exploitation IOSB, Interactive Analysis and Diagnosis (IAD), Karlsruhe, Germany; ^6^ FZI Research Center for Information Technology, Karlsruhe, Germany

**Keywords:** service robotics, healthcare robot, smart hospital, human-robot interaction, motion planning, robotic manipulation, no-code programming, deformable objects

## Abstract

Germany’s healthcare sector suffers from a shortage of nursing staff, and robotic solutions are being explored as a means to provide quality care. While many robotic systems have already been established in various medical fields (e.g., surgical robots, logistics robots), there are only a few very specialized robotic applications in the care sector. In this work, a multi-functional robot is applied in a hospital, capable of performing activities in the areas of transport and logistics, interactive assistance, and documentation. The service robot platform HoLLiE was further developed, with a focus on implementing innovative solutions for handling non-rigid objects, motion planning for non-holonomic motions with a wheelchair, accompanying and providing haptic support to patients, optical recognition and control of movement exercises, and automated speech recognition. Furthermore, the potential of a robot platform in a nursing context was evaluated by field tests in two hospitals. The results show that a robot can take over or support certain tasks. However, it was noted that robotic tasks should be carefully selected, as robots are not able to provide empathy and affection that are often required in nursing. The remaining challenges still exist in the implementation and interaction of multi-functional capabilities, ensuring ease of use for a complex robotic system, grasping highly heterogeneous objects, and fulfilling formal and infrastructural requirements in healthcare (e.g., safety, security, and data protection).

## 1 Introduction

Already today, nursing in Germany suffers from a severe shortage of skilled workers (Isfort et al., 2018). As in many countries worldwide, demographic change and the aging of society will further intensify this problem in the upcoming years. On the other hand, the nursing staff are already subject to high time pressure and high workload compression (Schmucker, 2020; Drupp and Meyer, 2020). The introduction of robot technologies to the healthcare sector has been discussed for some time as a way to relieve the workload of nursing staff. As robots are more and more present in hospitals in logistics and surgery (Okamura et al., 2010), the willingness increases to open up the nursing field for robotics. Nursing covers various tasks in changing dynamic environments with close physical contact with patients and others with nearly no contact with patients. It is possible to create robotic solutions specialized in single tasks or to develop multi-functional robots that can address a wide range of tasks. Moreover, it is important to distinguish between highly automated solutions with no human support and cooperative robot solutions working closely with the nursing staff.There are various works addressing these tasks and challenges in robotic nursing. In (Sayed et al., 2020; Maheu et al., 2011) robotic arms assisting restricted persons and in (Park et al., 2020; Perera et al., 2017) robotic solutions for feeding and serving food are presented. Surveys regarding robotic systems in the context of mobility and rehab for walking are presented in (Fritz et al., 2019; Bhardwaj et al., 2021). Furthermore, in (Liu et al., 2022; Yoshimi et al., 2022), lifting robots are presented to support the caregiver, and in (Christoforou et al., 2020), an overview of assistive robotics in care for older adults is presented. A robotic approach to empathy in nursing is shown in (Van Wynsberghe, 2013). Methods for safe and intuitive multimodal human-robot interaction are presented in (Abbasi et al., 2019; Zhao et al., 2023; Feng et al., 2022). In (Mišeikis et al., 2020), an approach is presented that is relatively similar to ours as it also deals with humanoid/multi-functional robots in a care context. Finally, in (Kriegel et al., 2022), the requirements and possible applications of robotics in hospitals are analyzed from the perspective of a nurse.These highly complex humanoid or service and assistance robots are of great interest as they can solve many different nursing tasks with one system. Their size and design are similar to humans; therefore, they are directly compared to the nursing staff regarding performance, flexibility, and interaction skills. The typical tasks expected to be solved by such robot systems are

• Guidance and/or transport of patients,

• Lifting and helping patients from bed to stand,

• Handling and delivery of known and unknown objects and material,

• Human detection, motion and speech recognition,

• Dynamic motion control and navigation in open environments,

• Remote control and avatar functions for telemedicine,

• Automatic documentation and connection to information management.

This work will focus on multi-functional service and assistance robots to support nursing tasks. There are several robots and research projects that have contributed to challenges in this field. They can be distinguished by the assistive domains concerning nursing care: assistance for nursing staff; work facilitation; assistance for patients with a focus on patient independence; rehabilitation; assistance with documentation and information management. Moreover, it is interesting what specific challenges the projects and robots address. The projects and robots, as well as their assitive domains and main functionalities, are presented in Table 1. More projects and robots can be found in the following survey articles (Khaksar et al., 2023; Martinez-Martin and del Pobil, 2018; Nieto Agraz et al., 2022).

**TABLE 1:** Overview of robots and projects for use in nursing, their assistive domain and key functionalities.

Robot or Project	Assistive Domain	Key Functionalities
GARMI (Geriatronics, 2021)	assistance for independence, work facilitation	telemanipulation, measurement of vital signs (remote)
Care-O-bot 4 (Kittmann et al., 2015)	assistance for independence, work facilitation	navigation, guidance, transport of objects and material
EVE (Research Council of Norway, 2023)	assistance for independence	guidance of patients, transport of objects and material
ROBINA Project (Rivera et al., 2019)	assistance for independence, assistance for nursing staff	handling of objects and material, remote control assistance
KURUMI (Terashima et al., 2022)	assistance for nursing staff, documentation	speech recognition, wandering persons with dementia
MKR (Muratec Keio Robot) (Terashima et al., 2022)	assistance for nursing staff, rehabilitation	guidance of patients, transport of patients and objects
Terapio (Tasaki et al., 2015)	work facilitation	transport of objects and material, guidance, speech recognition
FRIEND (Grigorescu et al., 2012)	assistance for independence, assistance for nursing staff	handling of objects, guidance of patients
RIBA (Mukai et al., 2010)	assistance for nursing staff	lifting of patients
PeTRA Project (Miller et al., 2023)	assistance for nursing staff	assistance for independence, transport of patients and objects
Pepper (Blindheim et al., 2023)	assistance for nursing staff	assistance for independence, rehabilitation
Lio-A (Mišeikis et al., 2020)	assistance for nursing staff, work facilitation	Handling of objects, guidance of patient

The goal of the HoLLiECares project was to develop a proof of concept for a multi-functional robot whose supporting tasks are relevant to nursing in hospitals. In contrast to most of the projects and works described above, the approach was not purely technology-driven but a co-design (Grindell et al., 2022; Vargas et al., 2022) with participating nursing facilities was applied emphasizing the integration into practical, everyday use and the social reality of clinical nursing. The contribution of this paper is the description of the further development process of the HoLLiE robot towards a care robot in a co-design process between stakeholders from the care sector and developers, as well as the proof of concept evaluation of the robotic prototype. As no standardized proof of concept evaluation can be found in the literature, the evaluation is based on the framework proposed in the work of (Elliott, 2021). The *prototype* mentioned there is the further developed HoLLiE robot, which demonstrated certain sub-functions in so called *proof of concept demonstrations*. The evaluation of these proof of concept demonstrations takes place within the use case descriptions (section 4) based on the question of whether the implemented sub-functions achieved the desired goals.

The structure of the paper is as follows: section 2 presents the development process of the use cases and the communication process with the stakeholders involved. Section 3 provides an insight into the further developed service robot HoLLiE, and section 4 presents the developed use cases in detail. Section 5 concludes with a discussion of the results.

## 2 Multi-step development process

The development should take place in the form of a co-design and explicitly include the perspective of nursing staff and other user groups in hospitals to specifically develop a robot that would not perform nice-to-have tasks but would instead take on necessary and relevant tasks within a nursing ward. A multi-step, iterative process was developed to determine the needs asynchronously and remotely, if necessary, especially given the first COVID-19 lockdowns that impeded the development. Originally, the development was to take place in on-site workshops at the participating hospitals. Due to the first COVID-19 lockdown in Germany in March 2020, the development was carried out online. The process mentioned above was developed as a pragmatic approach to enable especially the nursing practice partners to work asynchronously as much as possible, as the workload in the hospitals was very high, again due to COVID-19. Therefore, the nursing practice partners could not guarantee attendance at every online meeting. The process included the following steps:

1. Identification of requirements and development of an initial set of possible scenarios (initial hypotheses) by nursing science and nursing practice: 14 hypothetical scenarios were written by the nursing science research team. These initial scenarios were developed within a framework of 3 previously agreed fields of activity (transport and logistics; assistance in nursing care; documentation and information in nursing care), and then revised and supplemented by nursing practice. Revisions and requirements were discussed in an online workshop for each participating hospital. Participants were the research team (technology partners, nursing science partners) and the nursing practice partners of the hospitals. The focus of the discussion was the requirements in the respective hospitals. Scenarios were modified according to the discussions.
2. Initial assessment of this first set by the technology partners with regard to (technical) feasibility: Each technology partner received the scenarios developed in step 1. Results were discussed, and the scenarios were modified and refined in an online workshop with technology partners, nursing practice, and nursing science.
3. Prioritization of the first set by the nursing practice: The resulting scenarios from step 2 were prioritized by each hospital according to their importance. Prioritization was done asynchronously. The most prioritized scenarios should be developed further.
4. Further development of the prioritized scenarios by all partners in several online workshops until the 6 implementation scenarios were selected and consented to. Participants were the research team and representatives from different fields of the hospitals who were chosen and invited pragmatically by the nursing practice partners. Due to COVID-19 and the very high workload in the hospitals during that time, the number of participants varied from 1 to 8 persons from the fields of nursing management, geriatric medicine and nursing, pediatric nursing, medical wards, surgical wards, digitalization working groups, and staff units for the development of nursing care.
5. Concretization of the implementation scenarios with regard to technical aspects (interface, data flow, hardware, responsibilities): Technology partners used online workshops and 2 workshops in person (after COVID-restrictions were lifted).
6. Technical implementation.

Steps 1 to 4 can be seen as a co-design development process where nursing practice partners were involved in every step of the design and development process. Steps 5 and 6 concern the technical development and implementation of the scenarios. Additional visitations were organized for individual technology partners on specific aspects: a) wound documentation in each one of the participating hospitals. Researchers from a technology partner observed the process of wound treatment and documentation; b) spatial conditions in one hospital. A researcher from a technological partner explored and mapped the spatial conditions in an exemplary hospital ward. At a later date, after COVID-19-restrictions were lifted, an on-site workshop was held in each of the two hospitals, where the development status was presented to hospital staff. The workshops were open events. Whoever was interested could attend. Representatives from nursing, physiotherapy, occupational therapy, the ethics committee, IT, data protection, and administration took part.

## 3 The service robot HoLLiE

**FIGURE 1:**
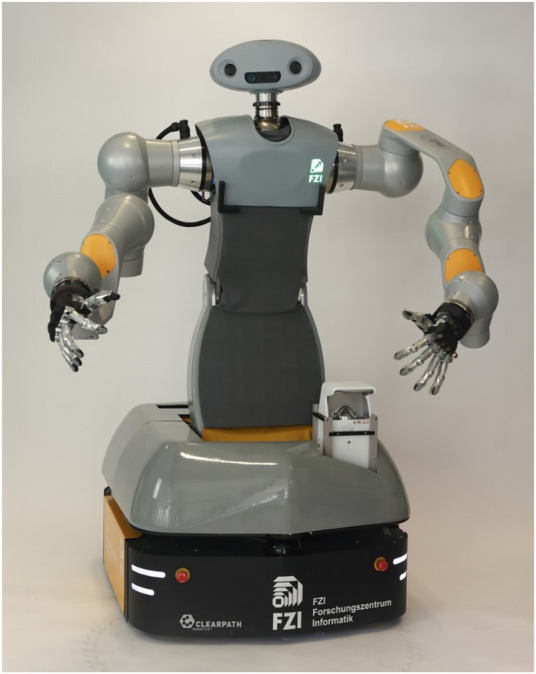
The bi-manual, ominidirectional robot platform HoLLiE, equipped with two laser scanners, a stereo sound system, a Kinect Azure, a short distance projector and two force-torque sensors.

The first version of the bi-manual robot HoLLiE (full name: House of Living Labs intelligent Escort) was completed at the FZI Forschungszentrum Informatik in 2011 (Hermann et al., 2013). Since then, the robot has been continuously improved. HoLLiE, as shown in Figure 1, has an actuated body, an omnidirectional mobile base, two laser scanners, speakers, and a multi-color LED badge in the chest. The actuated body is composed of two 6-DOF arms (Pilz PRBT arm) and two additional joints in the torso. In the picture, HoLLiE is equipped with two 5-finger hands (Schunk SVH), but as the hands can be changed according to the task, other hands were also used in the tasks addressed in this work. The arms have integrated joint position control circuits and do not require any additional controller boxes, making them ideal for mobile manipulation. With a weight of 135 kg, the Clearpath ridgeback platform offers a stable commercial mobile base where there is no danger of tipping over when moving the arms. The maximum speed of 1.1 m/s is appropriate for a hospital environment. In the course of HoLLiEcares, HoLLiE was additionally equipped with a kinect azure, a projector, a tablet, and two force-torque sensors (FTS) (Steffen et al., 2023). The technical improvements are motivated by the use cases described in section 4.

HoLLiE’s design enables easy maintenance of hardware components. By removing the gray magnetic cover on the mobile base, the majority of installed electronics can be accessed. The robotic system embodies three PCs, which are connected via Ethernet and communicate using ROS (Robot Operating System) (Quigley et al., 2009). Tasks are clearly distributed among the three PCs. The PC of the Ridgeback platform handles low-level coordination, communication, and control tasks. It provides the roscore and the movement of the mobile platform. A second PC handles mid-level tasks, sensor and actor communication. High-level tasks such as motion planning, navigation, mapping, and everything related to computer vision are implemented on a third PC.

## 4 The developed use cases

In HoLLiECares, a total of six use cases were selected and then implemented. The selection of the six use cases mentioned below is the result of steps one to four of the co-design development process described in section 2:

1. Pushing a wheelchair
2. Autonomous accompaniment of patients to examination rooms
3. Instructions for body movement
4. Wound documentation
5. Storing medicine
6. Handling of non-rigid objects

The following subsections describe the individual use cases in more detail.

### 4.1 Pushing a wheelchair

Walking exercises, which are carried out for remobilization, especially with older patients or after operations, usually require two caregivers. One person leads the patient, and a second person follows the patient, pushing the wheelchair for safety, so the patient can sit down at any time. Mobilization has a high priority as it is very important for recovery (Tazreean et al., 2022; Paton et al., 2018), however, as it requires two nurses at once, it is difficult to find enough time for it in the clinic’s daily routine.

#### 4.1.1 Why this use case?

In this use case, HoLLiE replaces a second person by taking over the pushing of the wheelchair. This use case is very suitable in terms of acceptance, as here the patient is not left alone with the robot; instead, a human is still present. From a scientific point of view, the motion of a coupled system in a dynamically changing environment such as a hospital with narrow corridors is an interesting challenge. This work addresses these challenges by providing an approach for whole-body motion planning for non-holonomic motions with a wheelchair, including the steering motions of the arms needed to adapt the turning radius of the wheelchair.

#### 4.1.2 Description of the use case and requirements

The walking exercises must start at the patient’s bedside, as these are immobile patients. The nurse helps the patient out of bed and into the wheelchair. When the patient sits in the wheelchair, the next step is to drive to a suitable start position. Ideally, a place that offers enough space and that is not heavily frequented. This is done by the caregiver who is better able to handle doors and achieves a smaller turning radius compared to the robot. However, depending on space conditions, this can also be done by HoLLiE.The nurse activates HoLLiE either via speech or manually using the scheduler. Next, the brakes of the wheelchair are locked and HoLLiE grasps the wheelchair in which the patient is located. The patient gets up with the help of the nurse who then releases the brakes again. As soon as the patient begins to walk, HoLLiE pushes the wheelchair to follow at a defined distance. Throughout the complete walking exercises the robot adapts to the pace of the patients. If they want to sit down, they can indicate this to HoLLiE via speech. However, if the patient spontaneously falls backwards, this must also be intercepted, therefore a close distance between patient and wheelchair is important. Therefore, a number of requirements can be derived from this use case. The robot must be able to grasp and steer the wheelchair in a dynamic and confined environment such as a hospital. The planned motions of the coupled systems must allow the robot to turn with a minimum turning radius. The patient’s distance from the wheelchair must be monitored and controlled during remobilisation walking exercises. And the occlusions created by the wheelchair and the patient must be handled by the navigation system.

#### 4.1.3 Description of the developed subsystem

For the implementation of the use case, path planning, control and collision avoidance of a coupled robot system with non-holonomic properties is required. In the current state of the art, there are only a few approaches for pushing a wheelchair (Nozawa et al., 2008) or similar objects (Scholz et al., 2011; Rioux and Suleiman, 2018) by a robot.

For the scenario the existing navigation stack of HoLLiE, see (Steffen et al., 2023), was extended. Thereby, a Timed-Elastic-Bands (TEB) baed planner was implemented (Rosmann et al., 2013). The TEB was extended to be able to plan smooth trajectories taking into account the coupled kinematics of HoLLiE and the wheelchair (see details in (Schulze et al., 2023)). HoLLiE’ mecanum-wheeld base is holonomic, thus, capable of moving sideways and diagonally as well. However, by pushing a non-holonmic wheelchair, the degrees of freedom (DOFs) are restricted. Therefore the combined system must be described by an inverse Ackerman kinematic. In addition, the use of the wheelchair necessitates more complex turning maneuvers. It demands higher requirements for a smooth ride and the consideration of a larger turning radius. The TEB, which serves as the basis for the approach, plans reactively up to an adjustable time horizon. The Levenberg-Marquardt method is used to optimize the trajectories.

The extended TEB considers the geometry of the wheelchair (Schulze et al., 2023). In addition, new constraints were introduced to allow optimization with respect to “softer” trajectories and less centrifugal forces. The following constraints were introduced: translational velocity, angular velocity, translational acceleration, angular acceleration, global path duration and obstacles. All of the above constraints were applied separately to the Clearpath Ridgeback platform and the wheelchair, except for the global path duration. The comfort of the patient is important because it directly affects the appropriation of the system. To determine comfort, the path is recorded and its rate of acceleration change is analyzed. However, since there is no threshold at which a comfortable path can be distinguished from an uncomfortable path, a comparison was made with the acceleration curves achieved the reference planner.

**FIGURE 2:**
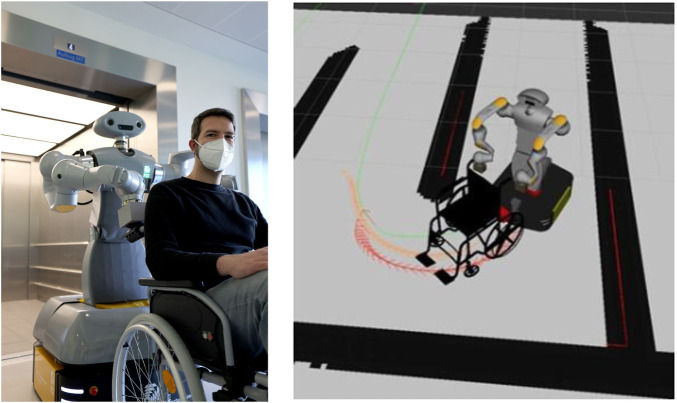
HoLLiE with a wheelchair in the Städtisches Klinikum Karlsruhe (left). In the robotics simulator gazebo it is shown how HoLLiE pushes a wheelchair around a tight curve. The position of the arms is very important for this task (right).

In tight curves, as shown in Figure 2, it is crucial that the robot’s arms do not remain rigid but move according to the displacement while rotating the wheelchair. This motion significantly reduces the turning radius of the wheelchair. To achieve this, the extended TEB introduces as an additional parameter: the position angle β . It indicates the exact orientation of HoLLiE relative to the wheelchair. This position angle is necessary for the arm control during pushing. Without the implementation of the arm controller, HoLLiE would not be able to push a wheelchair in real-world hospital corridors as it would then only be able to make very wide turns. The software subsystem of the arms receives the position angle β as input and uses this to determine the gripper poses. Therefore the real-time capable dual arm controller uses the current target angles from the extended TEB planner and translates them into joint commands to ensure accurate and synchronized movements of both arms. The resulting outputs are the individual joint positions of both arms changing over time.

Another important component is the detection and localisation of the patient, her or his legs and the wheelchair in the sensor data of the robot. The wheelchair and the patient’s legs strongly obscure the frontal laser scanners’ view of the mobile base. Without prior filtering, the mapping and the adapted TEB planner would therefore constantly register the wheelchair and the person as obstacles. Since the point clouds captured by the Azure Kinect in the head of HoLLiE also contain the wheelchair, they must be removed by a filter. To detect a possible user, a region of interest (defined as a rectangular bounding box) is placed around the coordinate frame of the wheelchair. To ensure that the bounding box is always in the correct position in real time, the position of the wheelchair is determined by proprioception. Thus, the pose of the wheelchair frame must be determined directly from the angle and the assumption that the wheelchair and the grippers form a fixed link. Since also the arms of HoLLiE can be in the field of view of the Azure Kinect, an additional self-body filter package is used to filter out the geometry of the arms and grippers. The approach is adapted from (Scholz et al., 2011) and overall the following filters were implemented for the final application:

• HoLLiE body filter: surface points of the arms and hands from the point clouds.

• Wheelchair filter: filters surface points of the wheelchair and potential passengers through a simple bounding box

• Ground filter: all points with z-value < X are ignored

• Reflection filter: Based on intensity (laser) and statistis

The extended TEB approach was thoroughly evaluated in a number of challenging virtual environments using a ROS-based simulation. The proposed approach showed good performance in narrow corridors and challenging environments. Details of this extensive evaluation are shown here (Schulze et al., 2023).

#### 4.1.4 Real life tests

**FIGURE 3:**
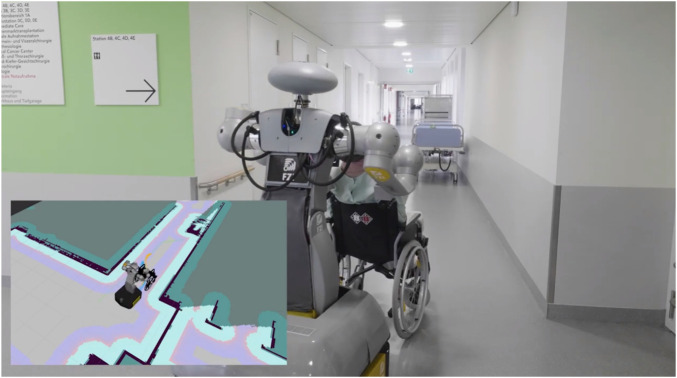
HoLLiE pushing a wheelchair around a curve in the Städtisches Klinikum Karlsruhe. The planned path is displayed on the real occupancy grid map of the hospital station.

The entire use case ’pushing a wheelchair’ was tested during the second real-life tests at the Städtisches Klinikum Karlsruhe (SKK), as shown in Figure 3. The navigation with the wheelchair worked well on the software side, which can be seen by the results of the simulation tests published in (Schulze et al., 2023). However, there were some integration challenges during the transfer to the real hardware. Previous tests in the lab had already indicated that autonomous navigation of the coupled system is possible in general, but several major issues remained. Curves exert a lot of shearing forces on the robot’s actuators, which is massively aggravated by the weight of a patient. Hence, the real tests were not carried out on patients but on a mannequin from the nursing school with less weight. Although some curves were successfully driven with a light load, the impact on the robotic hardware was enormous. This resulted in damage to the custom designed 3D-printed grippers. As a result, the real hardware tests were stopped at an early stage because of the high risk exposed to HoLLiE’s arms.

#### 4.1.5 Results and lessons learned

One major issue for the relatively poor practical results on the real robot is that the arm control is not sufficiently reactive and provides no compliance at all. Therefore, no feedback from the force sensors was taken into account by the control approach. Thus, even little bumps and disturbances caused by slightly uneven ground jammed the mechanics. As the planner still continued to steer, this quickly lead to hardware overload and would have lead to damages. A key finding is, that real world applications require compliance to deal with non-optimal environments. Also the size of the turning circle still caused practical limitations, even as it was minimized by exploiting arm motions. This worked out in simulation but was not feasible with a real wheelchair due to the friction of the front wheels. The planner experienced problems when performing more complex turning maneuvers, like a car with a trailer. In these cases it preferred rotations in which the wheelchair remained fixed and HoLLiE drives a semicircle around it. It has been shown that for this use case it would be helpful to incorporate feedback from force torque sensors or compliant arms into the overall control and motion planning approach for such a coupled system. As the wheelchair motion controller is based on an optimization loop that runs at 50 Hz, a penalty signal could be added to the optimization term to indicate error situations. Alternatively, a vector or local ’field’ could be calculated to indicate which direction will cause issues. After all, feasibility could be demonstrated for the highly challenging task of driving a wheelchair through a hospital via a holonomic bimanual service robot. Also important scientific progress was achieved concerning the arm motions that are required to drive sharp turns.

As part of the proof of concept demonstrations the implemented sub-functionalities of this use case were divided into the coupling based on the grasp and steerability of wheelchair, the trajectory planning for the coupled system created by the robot and wheelchair, manoeuvring the wheelchair with a mannequin and also pushing the wheelchair with a human. The connection of the robot with wheelchair handle, the resulting steearability and the trajectory planning for the coupled robot and wheelchair were very successful in the simulation, lab experiments and real world tests. Unfortunately, pushing and manoevering the wheelchair with a mannequin only partly worked out and the test with pushing a human in the wheelchair did not work out at all. The main reasons where the high torques and forces resulting from the coupled wheelchair on the arms, especially with a patient sitting in the wheelchair. But as proof of concept evaluation, the coupled trajectory planning between robot and wheelchair was the core component of this use case. This was successfully implemented and demonstrated on site. For the practical, everyday implementation of this use case, a more specialized, compliant robot is needed instead of the HoLLiE robot, which served as test and development platform in this work.

### 4.2 Autonomous accompaniment of patients to examination rooms

**FIGURE 4:**
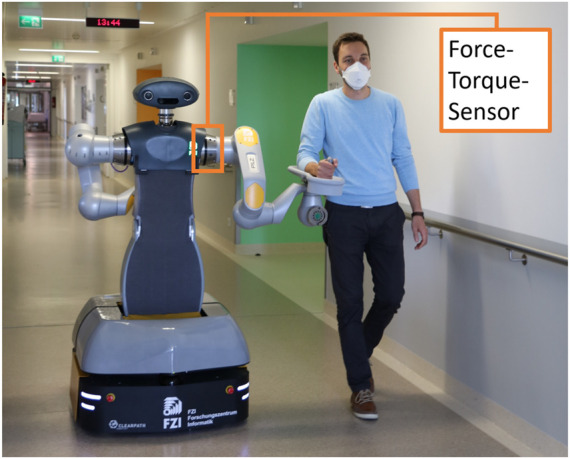
HoLLiE robot in the Knappschaftsklinikum Saar (KKS) accompanying a human.

In this use case, the HoLLiE robot accompanies and supports patients to examination rooms (see Figure 4). No additional nurse is required.

#### 4.2.1 Why this use case?

Patient mobilization contributes to patient’s recovery (Ferris et al., 2020). Therefore, we conclude that accompanying patients that are able to walk to an examination room in a walking manner is better than pushing them in a wheelchair. When they are able to walk completely independently without support, they only need to be shown the way. If, on the other hand, they need physical support, the robot must be able to support the patient. By having a robot take over such a physically supported escort, full consideration can be given to the patient’s desire for speed. The advantage of a robot is that it is not in a hurry, as nurses often are today (Govasli and Solvoll, 2020). At the same time, nurses are relieved of time and physical strain, especially when traveling long distances to examination rooms. This use case is therefore equally beneficial for patients and nurses.

#### 4.2.2 Description of the use case and requirements

In this use case, the patient is to be picked up in his or her room. Patients who, for example, cannot yet walk safely without support after leg surgery should be able to support themselves on the robot. In addition to the support, the robot shows the way to the examination room. No leader-follower concept should be used, in which the robot specifies the exact trajectory (geometric path as well as speed), but the human should be able to influence the choice of speed and also the exact path, e.g., around a curve. Thus, an emancipated concept shall be implemented. This requirement results from a video analysis of assisted accompaniment of patients by nursing staff. Here, nonverbal haptic communication and agreement through the exchange of interaction forces between the patient and the nurse for trajectory selection was observed. This nonverbal communication in the form of the exchange of interaction forces is also to be replicated on the robot.

#### 4.2.3 Implementation of the use case requirements and description of the developed system

From the state of the art and research, no existing application was known that combined emancipated guidance with haptic support. Therefore, a new functionality had to be developed for accompanying a patient to examinations, which required on the one hand a hardware extension of the robot and on the other hand the development of a local path planning software that takes into account the human’s motion request.

For the hardware implementation of the use case, force torque sensors were first added to the shoulder joints of the robot in order to be able to measure the interaction forces between the robot and the patient. In addition, a forearm rest was developed on which the patient can rest. By developing a new joint housing cover, the forearm rest could be firmly attached to the robot arm. The height of the forearm rest can be adjusted and customized by rotating the arm joints.

For the software implementation, a local path planner was developed, which is published and described in detail in (Schneider et al., 2022). From the measured interaction forces, a local planner continuously estimates the human’s desired motion in the form of a motion primitive with longitudinal velocity component v and angular velocity component ω . The human’s motion desire is then compared to the robot’s desire motion primitive. The Base Local Planner (Marder-Eppstein and Perko, 2023) available in ROS was used to generate the robot’s motion primitive. An agreement on a joint motion maneuver is then determined from the two available motion desires based on negotiation. This approach is based on the model for emancipated agreement finding between a human and an automation presented in (RothfuÃŸ, 2022). This approach had to be extended to include a temporally repeated or continuous agreement process. In addition, the time-based negotiation deadlines used in (RothfuÃŸ, 2022) to ensure agreement finding had to be replaced by a behavior-based strategy because, in contrast to the application considered in (RothfuÃŸ, 2022), there are naturally no deadlines in cooperative trajectory finding. In the present case, agreement finding was done via the implementation of a novel reciprocal tit-for-tat strategy. The local trajectory planner was implemented as a local trajectory planner plugin for the Move-Base-Flex navigation framework (Pütz et al., 2018) available in ROS.

#### 4.2.4 Real life tests

In an initial test in the two clinics involved, the basic functions for accompanying a patient by remote-controlled escort were first tested. This included measuring the interaction forces with the force torque sensor and checking the height and shape of the forearm rest for patient comfort. In particular, the stability of the forearm rest was then reinforced and it was mounted lower on the robot arm in order to accompany smaller patients in particular. In the second real life test, the overall functionality was to be tested. Unfortunately, due to various circumstances (especially hardware issues and unexpected integration issues) the full functionality of accompanying a patient could not be put into operation in the end. However, in (Schneider et al., 2022) the simulative results of the cooperative trajectory planner can be found.

#### 4.2.5 Results and lessons learned

In order to evaluate the results and success of this use case, the use case is divided into three sub-functions. The first subfunction is the haptic support device for the patient on the robot which was successfully developed and implemented on the robot. The second sub-function is the cooperative trajectory planning which worked successfully in simulation. From the scientific point of view, this development of a cooperative, emancipated trajectory planner is the main result of the use case which was published in (Schneider et al., 2022). The third sub-function cooperative trajectory planning in reality integrated on the robot, which could not be shown. Even though real-world validation is still pending, it can be said that the proof of concept has been demonstrated with the simulative results in (Schneider et al., 2022).

The main experiences from the development of the application are that patients were very open and interested in this type of application and were happy to be accompanied by a robot for the tests. This is in contrast to the statement of some nurses that during the accompaniment of patients to examination rooms very often important interpersonal moments between patients and nurses take place (patient reveals fear of examination or confides in a nurse after a doctor’s visit). In these moments, the nurse exerts empathy and compassion towards the patient. This is not possible for a robot. Consequently, in this application in particular, a very good balance must be found between the question of what is technically feasible and the ethically reasonable and helpful use of technology.

Challenges in implementing this use case were primarily passing doors and elevators that could not be opened remotely. Another challenge with regard to passing through doors was the fact that the HoLLiE robot was further developed from an existing robot platform that was not originally developed specifically for use in a nursing ward. This meant that the robot base and shoulders fit through a door, but were actually too wide in combination with an accompanied patient. Secondly, the further development of the existing system had the disadvantage that the available arms installed on the robot were not designed to bear a full body weight of around 80 kg. A load of 20 kg was considered as the upper limit for the tests. Other existing challenges include health emergencies such as vertigo. One possibility here would be an emergency remote call to a nurse, who can then talk to the patient. A difficulty remains the possible fainting of a patient, as it is not yet possible for a robot to support an unconscious patient within a fraction of a second. The safety aspect of autonomously accompanying a patient therefore remains a major challenge. Finally, process safety is also an unresolved issue. If, for example, patients decide halfway to the examination room that they do not want to go for the examination, they could simply flee from the robot. A human nurse could respond to the patients, talk about their concerns and help them to make a well-considered decision.

### 4.3 Instructions for body movement

#### 4.3.1 Why this use case?

In this scenario, the robot shall support the clinic staff by guiding patients through movement exercises. The movement exercises are physical exercises to activate the circulation of the patient and prevent thrombosis, and finger exercises to maintain finger mobility. The support for the clinical personnel in this use case consists mainly of time savings. During the scenario selection stage the nursing staff informed us that ideally, the exercises for thrombosis prophylaxis would have to be performed every 30 minutes for at least 2 minutes. However, patients often forget them. The functionalities implemented in this use case are modular and can also be installed stationary with the help of a camera and computing unit. The advantage of using a mobile platform however is that it is not tied to one room and not every room has to be retrofitted with its own system. As the detection components are only dependent on hardware that is usually already installed on the robot anyway, this use case also offers added value without the need for retrofitting hardware. In addition, this scenario is not time critical and can be scheduled during charging breaks in which the robot is needed to keep stationary.

#### 4.3.2 Scenario description

The following sequence was defined for the implementation of the scenario. Since the robot could not drive through the door frames of the clinics without manual control due to its size, the scenario begins directly in the patient’s room. The patient is already in bed. The robot positions itself either at the left or right end of the bed so that its tablet points in the direction of the patient. In a friendly voice, the robot introduces itself as HoLLiE and explains that it will now guide movement exercises that are also displayed on the tablet screen. The exercises are guided by voice output and a visualization via a tablet attached to the robot. HoLLiE then asks, “Are you ready?” Once the patient agrees, the robot begins to instruct body movement exercises. The question was aimed at ensuring that the patient could start the exercises at a time that suited them. We assumed willing patients for the tests as they had to give their consent prior to the test. If they said no, the instructions would have been aborted. A suitable strategy for unwilling patients in everyday hospital life would have to be investigated in further studies. The body movement exercises take place while the patient is lying down. A camera-based evaluation of the exercises is used to detect and point out errors during exercises. In case of pain the scenario shall stop immediately. After completing these exercises, HoLLiE guides the finger exercises. There are a total of eight hand exercises and eight body exercises. The instructions assume that a physical therapist has performed the exercises with the patient at least once and that they are understood. Finally, HoLLiE thanks the patient and says goodbye.

#### 4.3.3 Description of the developed subsystem

The hardware needed for this scenario are a Kinect Azure depth camera for the person detection which was mounted on the robot head, a 10-inch Android tablet that was mounted on the robotic chest for visualization and configuration of motion exercises and a microphone for speech recognition. The internal tablet microphone was used since it was also needed in the wound documentation scenario and the tablet was detachable (see section 4.4). The speaker of the system is located in the chest of the robot. All software components are developed using the ROS framework.

**FIGURE 5:**
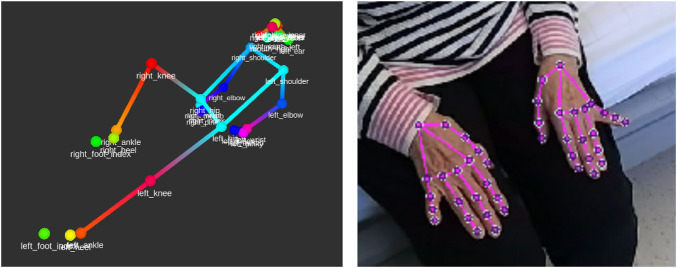
Visualization of a 3D body detection result and a 2D hand detection result.

The recognition of body exercises was implemented by a recognition of joint angle states. The lifting of the arm is recognized by the fact that the angle between the upper body and the arm is large and, in addition, the arm ideally has an angle of 180°. The choice of this ontological classification has two main reasons. First, it makes it possible to add new exercises without extensive data acquisition and training of a classifier by specifying the necessary joint angles. In addition, it was important to physiologists to obtain angles to identify any improvements or deteriorations in the patient’s condition. Since angles cannot be estimated from the obtained 2D skeleton on the color image, a 3D skeleton of the patient was inferred from the cameras depth image (see Figure 5). The ideal position for the robot during the movement exercises turned out to be the foot end of the patient’s bed, since looking at the tablet on the side of the bed for too long would lead to an unfavorable neck rotation.

For person detection Mediapipe (Lugaresi et al., 2019) was chosen because this method runs in real-time on a PC without a GPU which was found suitable for a battery powered robot. In addition, Mediapipe can also detect hands and determine the hand pose (see Figure 5). Since Mediapipe only detects one person in an image, the object recognizer YOLOv5 (Jocher et al., 2022) was used as a procedural optimization. YOLOv5 detects objects and persons and returns their bounding boxes. Body pose estimation was then performed on the bounding boxes of all detected persons. This approach also allows optimization to discard irrelevant individuals if a specific person is to be tracked.

As described, a person and his hands are captured as a 3D skeleton. If an angle is defined for one or more joints, this is called *configuration* in the following. An exercise consists of a sequence of skeletal configurations. For example, the exercise *lift arm* consists of the configurations *arm up* and *arm down*. The required joint angles were determined empirically from the recordings of the Wizard-of-Oz experiments conducted in the hospitals.

**FIGURE 6:**
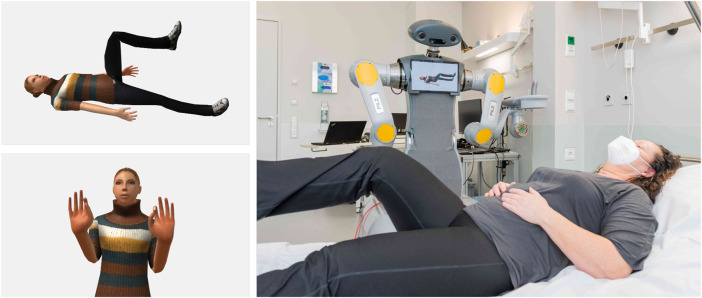
Visualization of a body and a hand exercise (left). Performing body exercises with a patient (right). Source photo right: Markus Kümmerle, Städtisches Klinikum Karlsruhe (CC BY-SA 3.0).

The exercises were visualized using the tablet installed on the upper body of the robot. An avatar was animated on the tablet to demonstrate the exercises (see Figure 6). It was found that patients mostly ignored the voice instructions in case of execution errors and only imitated the visualization. Auditory instructions for correct execution of the exercises were also ignored. Cues that an exercise was being performed with the wrong half of the body were not perceived. Therefore, dimming the visualization when exercises were repeatedly performed incorrectly needed to be implemented to direct the patient to the voice instructions.

**FIGURE 7:**
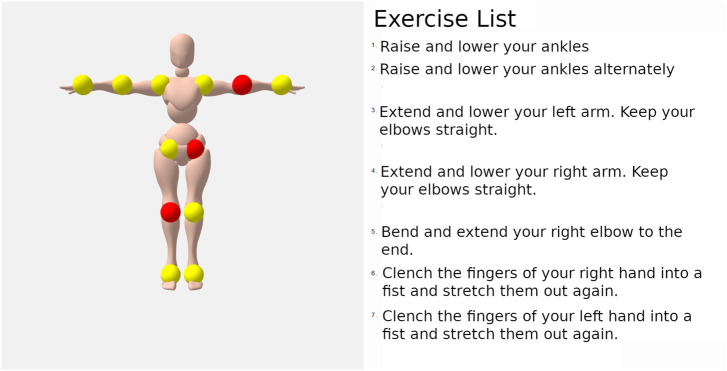
Joint selection for exercise composition.

For the body and hand exercises, a standard sequence of exercises was specified by the physiotherapists of the KKS in Püttlingen. A web GUI for the tablet (see Figure 7) was implemented via which individual joints that should not be loaded can be excluded by tapping on them. A neutral 3D avatar was chosen to select the exercises to be performed.

The implemented voice interaction during the movement exercises is limited to questions and answers related to the exercises. For speech input, the cloud-based speech recognition system from Nuance1 was used. This recognizes natural language and estimates the intentions of statements. To do this, sample sentences such as “Am I doing this right?” must be stored with a corresponding intention. The software also takes sentence structure into account and recognizes rephrasing. In the case of the question “Is this correct?“, the correct intention is also recognized. Overall, repeating the description and answering whether the exercise is performed correctly were implemented, as they had proven to be important in the Wizard-of-Oz experiments with patients. In addition, stopping the exercises if pain was suspected was implemented. The voice feedback alerted the patient when an exercise was performed incorrectly. The most common error in this case was confusing left and right.

#### 4.3.4 Real life tests

The evaluation of the overall system took place in two phases, each during real life tests in the clinics. Since robotic functions were not yet available during the first real life test, Wizard-of-Oz experiments were conducted instead to record video of patient reactions and interactions. Since the experiment was also designed to study how patients interact with the robotic system, a voice synthesis capability of any free text was also included to allow the robot to answer any questions during the Wizard-of-Oz experiment to maintain the appearance of an autonomous system.

For the experiments, the patients were informed verbally and in writing about the study and the institutes involved and had to give their consent. They were also informed that they could withdraw at any time without reason and without fear of consequences, and were also informed in writing by the data protection officer if they wished to have their data deleted at a later date. Due to the high level of patient fluctuation, suitable patients were selected by the nursing manager during the morning ward round and informed by us. When selecting the patients, care was taken to ensure that they were able to carry out the movement exercises. If they were in pain, the test would have been stopped immediately, but this did not happen. In addition, there was always a physiotherapist in the patient’s room during the tests.

In the experiment, a total of 18 patients were recorded in both clinics while performing physical and finger exercises in order to be able to optimize the acquisition components to be integrated afterwards. Recordings were made from different angles to investigate the influence of occlusions.

During the first real life test, the performance of the used algorithms was already assessed on site. At the time, however, these were not yet being carried out on the robot, but on another system. However, the camera for capturing the patient was already mounted in the robot head. The voice output was done via a wireless speaker which was placed on the back of the HoLLiE base and was connected to the computer of the experimenter.

In real life test two, the scenarios were then integrated completely on the robot. The technical components of Fraunhofer IOSB were completely integrated on HoLLiE and the scenario was successfully tested with two patients autonomously.

#### 4.3.5 Results and lessons learned

An assistance system has been successfully developed that is capable of visually and auditorily guiding patients through movement exercises. It recognizes and communicates physical exercises, hand exercises as well as any errors in the execution. For this purpose, a holistic detection of the persons, their activities and speech was implemented and integrated into an existing mobile robot platform and therefore fulfilled the proof of concept. Furthermore, the results of the person detection as well as the interaction modalities were made available to the other project partners for use in their scenarios. Due to the modularization of the developed components and the data management by means of the ROS framework, the functionalities can be easily reused in other research projects as well as extended by further components. As part of the proof of concept demonstrations the implemented functionalities were validated on real patients during the real-life tests at KKS and SKK. The tests covered the three main components: audiovisual instruction, voice communication, and the exercise detection. While the audiovisual instruction and the exercise detection components were successful in the tests, the speech recognition proved to be too inflexible. On the one hand, the sample sentences for the intention detection were too rigid and on the other hand, the dialects of some of the test subjects were too strong. Currently Large Language Models like ChatGPT (OpenAI, 2024) would be used to infer intentions.

### 4.4 Wound documentation

#### 4.4.1 Why this use case?

The complex demands of wound management are associated with a high time commitment for nursing staff. Due to demographic changes, there are more and more patients with chronic wounds. This also increases the documentation effort for the nursing staff. The study of Guest et al. (2020) describes the total number of wounds (prevalence) in the years 2017 and 2018 in the United Kingdom as well as their costs to UK’s National Health Service in these years. It highlights the increasing prevalence of chronic wounds due to an aging population and the associated costs and resource use in the NHS. At the same time staff turnover is high and the number of nurses is decreasing (Martin et al., 2023). This leads to a lack of time for patient care and therefore for documentation. The project analysed wound documentation processes and identified areas for improvement. Wound management provides a good basis for a digital assistance system. The documentation is structured according to the established wound care procedure. It was found that speech-based wound documentation can have a significant impact on reducing the workload of nursing staff, particularly in terms of ease of use and time savings.

#### 4.4.2 Description of the use case and requirements

This use case takes place in the patient’s room as part of wound care. HoLLiE provides access to the digital wound documentation via the web interface of the integrated tablet. Nursing staff can operate the patient file by touch or voice input. Context-sensitive speech recognition allows relevant wound care information to be digitally documented via voice input. The tablet can also be used to take pictures of the wound and store them in the documentation. Nurses can check and manually correct or add to entries.

#### 4.4.3 Implementation of the use case requirements and description of the developed system

To implement the speech-based wound documentation system, a speech processing component (speech service) and a user interface are essential.

##### 4.4.3.1 Speech service

**FIGURE 8:**
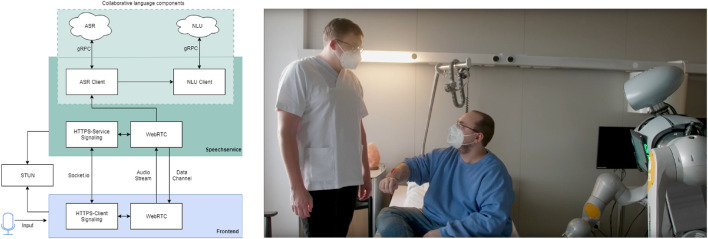
Collaborative language components (left) and image of the use case with speech-based, automated wound documentation (right).

The speech service consists of several collaborative components (see Figure 8 for the system architecture). Automatic Speech Recognition (ASR) first converts the user’s speech into text. Then a Natural Language Understanding (NLU) service extracts context and core information from the ASR texts. This is done using language models and lexical extensions from the field of wound documentation. As a basis for semantic interpretation, sentence structures are divided into intents and entities. An intent describes the superordinate meaning of one or more sentences (e.g., “wound size”). Entities contain the core information of the meaning (e.g., “length 10 cm”, “width 4 cm”, “depth 1 cm”). Entities can consist of several literals. A literal can be described by different words, such as ’sepsis’ and ’septic’. For more complex sentence structures with multiple entities, a composition is used. The wound width, for example, consists of the entities “unit of mass” and “wound width”. This allows the wound width to be expressed in millimetres and centimetres and the speech service to understand their relationship. The connection to the voice service is made via a web-based frontend. This means that the wound documentation can be used on all mobile devices. The microphone of the device is used as an input device. The connection is established, the audio stream is transmitted and the result of the voice service is sent back in several phases. In the next phase, a peer-to-peer connection is established and the audio stream from the microphone is transmitted to the voice service using the WebRTC communication standard. The stream is pre-processed within the voice service. The WebRTC component forwards the processed audio stream to the collaborative speech components. The result of the language components contains all intents with the associated entities and literals, as well as additional information. The evaluation logic in the frontend reads the intent and the values of the literal and updates the corresponding elements in the user interface (UI).

##### 4.4.3.2 User interface

The design of the user interface is based on the electronic wound management system currently in use at SKK. In a predefined structure, the nursing staff can document relevant information on wound healing and the measures taken. The User Interface was adapted for the prototypical implementation of the speech-based wound documentation system. The complexity of the system was reduced by removing description fields. The nomenclatures and operating elements of the UI were adopted in the prototype.

#### 4.4.4 Real life tests

The system was tested with 22 nurses from the KKS and the SKK. After a short briefing, the nurses used the system to document artificial wounds on a torso. Following the tests, interviews were conducted with the participating nurses. The qualitative expert interviews without a defined questionnaire provided realistic insights. The participants were asked questions about how much time they currently spend on wound documentation, whether they have used voice-controlled documentation systems to date and how much time they would estimate they would save by introducing such systems. Furthermore, an open discourse with users about the use of speech-based documentation in hospitals was initiated.

#### 4.4.5 Results and lessons learned

The speech-based, context-sensitive wound documentation system was developed and tested in a hospital environment. In particular, the high requirements of the existing IT infrastructures, the different user groups and the wearing of medical masks pose challenges for the technical implementation. The evaluation showed that intelligent documentation systems are highly relevant to the healthcare system and have great potential for use and time savings. This relevance was highlighted by the example of time-consuming wound documentation. During the course of the study, 15 of the 22 participants estimated the expected time savings from the use of intelligent documentation systems. Six of these 15 participants expected to save 10 min per wound. One person expected to save more than 10 min per wound, while 7 expected to save time but could not quantify it. One person did not expect to save any time with the proposed solution. The transferability of voice-based documentation systems to other areas was also considered with experts during the evaluation. In summary, the need for intelligent systems to reduce the administrative burden and the workload of medical staff was confirmed. However, the creation of large and heterogeneous datasets with domain-specific terminologies is essential for a comprehensive and promising use, especially of speech-based documentation systems.

Overall, the use case for wound documentation fulfilled the proof of concept. However, individual technical components showed the need for improvement for optimized productive use in the hospital. The speech-to-text component (ASR) used was robust enough for a proof of concept, but would still have to address certain problems for productive operation. In rooms with a lot of ambient noise, for people with pronounced speech dialects or when wearing masks, the results of the speech-to-text service were not always completely accurate. The NLU module was able to achieve good results for the defined test cases, but would also need to be trained for all marginal cases using a larger data set. In the case of several parallel conversations in the same room, it was also unable to differentiate between the different conversation parties, which could lead to problems with assignment. Heterogeneous datasets, which include a variety of data types and sources, enhance the ability of machine learning models to generalize across different contexts and terminologies. This is particularly crucial for domain-specific applications, as they require tailored linguistic and contextual understanding to achieve high accuracy and usability in real-world scenarios.

### 4.5 Storing medicine

#### 4.5.1 Why this use case?

One of the tedious daily tasks in a hospital is the restocking and screening of the pharmaceuticals in almost every department: Checking for missing items or for drugs that are over their expiration data and restocking them in an order, so that the longest lasting packages are in the back of the stock. As the task does not require any work with patients it is suited for automatic execution and can easily be scheduled during night shifts. This saves precious time of the personal, otherwise consumed by dull repetitive tasks.

#### 4.5.2 Description of the use case and requirements

The challenges of this scenario on the other hand are that the storage facilities vary in every department, the robot has to deal with a variety of container types and must operate in tight spaces. Therefore one essential requirement was, that the robot must offer efficient and intuitive interfaces to be reprogrammed for the local settings by non-robotics experts, namely, the nursing staff in the hospitals.

**FIGURE 9:**
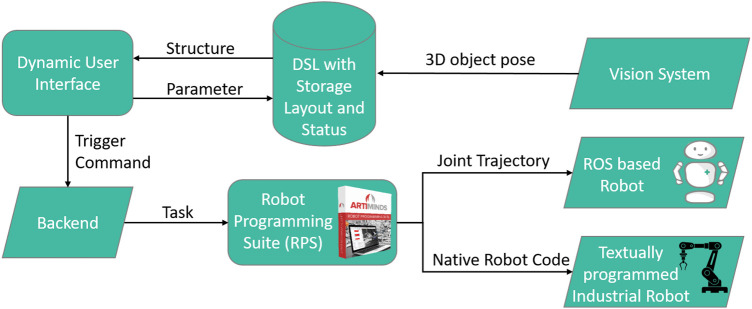
Meta programming approach with a DSL (Domain Specific Language) and an abstract robot task model (ARTM).

The steps to solve by the robot for this scenario consisted of a visual identification and 3D localization of medicine boxes of varying geometry, the opening and closing of drawers and the picking or placing of boxes. One aspect that was explored during the development was the exploitation of possible synergies between industrial use cases and the hospital/caring scenario. Some of the core functionalities and lots of robotics know-how is already used in commercial automation projects. The care sector must harness that fact and benefit from it. Therefore large parts of the implementation were realized in a portable way by using the industry proved ArtiMinds software stack (see Figure 9). It allows to shortcut many development efforts and to test the approaches not only on the HoLLiE robot but also on a textually programmed industrial robot.

#### 4.5.3 Hard- and software stack of developed subsystem

For the 3D localization and identification the onboard Microsoft Kinect Azure camera (Tölgyessy et al., 2021) of HoLLiE was used. This camera could be panned and tilted to focus on the workplace or the storage and delivered a high quality 2D RGB image as well as a 3D pointcloud. For the box manipulation we relied on a vacuum gripper manufactured by SCHMALZ, that was capable of reliably holding and lifting the smooth surfaces of the medicine boxes. Additionally a 3D printed hook was mounted next to the vacuum gripper that was used to open and close the drawers and the cabinet door. For the industrial setting a UR10e robotic arm manufactured by Universal Robots was used with a custom made vacuum gripper, the onboard force-torque sensor of the arm, but no camera system.

The software stack is built around the “ArtiMinds Robot Programming Suite” (RPS), which is used to plan collision free motions for a large number of different robots and translate from an abstract graphical program representation to robot specific programming languages. The core concept of the implementation is a so called three leveled “Meta Programming” that facilitates a “Domain Specific Language” (DSL) (Deursen et al., 2000) which is made to describe the storage layout and compartment geometries (Moreno Garcia, 2022). This DSL is used to dynamically create a user interface that aligns with the departments storage structure but also to automatically derive an abstract model of a robotic action-plan in the RPS’s template based graphical programming. The RPS then plans a concrete motion to achieve a pick- or place-task with the specific part coordinates and compiles it either into native industrial robot code or into a joint trajectory, suitable for ROS (see Figure 9).

The stack used for this in HoLLiE Cares consists of the RPS, running on an external Windows machine and three ROS nodes running on HoLLiE’s OS: One ROS node is a neuronal network for image segmentation (a YOLOv5 detector (Jocher et al., 2022)) used to classify and localize the medicine in the 2D images streamed from the Kinect camera. The resulting IDs and rectangular 2D bounding boxes were transmitted to the second node. It implements a 3D box fitting algorithm in the PointCloudLibrary (PCL) (Rusu and Cousins, 2011) which processes the 3D pointsclouds from the Kinect camera. As a result the algorithm delivers grasping poses in the center of the medicine box top surface. This information is then sent to a command-and-control node that passes it via a TCP REST (“Representational State Transfer”) interface to the RPS. After planning a collision free pick or place motion, the RPS sends a joint-trajectory back to ROS that deliveres it to HoLLiE’s ROS-control interface for execution (see Figure 9). Parts of ROS interface build upon previous research project result from the ROPHA research project (Alt et al., 2020). A second functionality of the command-and-control node is the visualization of the dynamically layouted user interface.

#### 4.5.4 Real life tests

**FIGURE 10:**
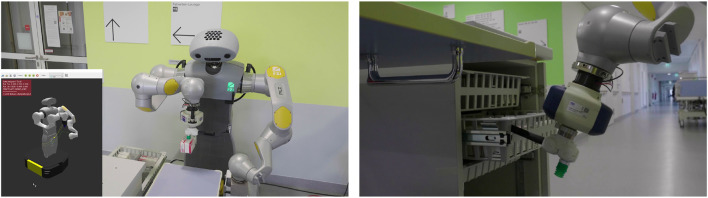
HoLLiE fetching a medicine box from a drawer and putting it on the table. Small picture shows the planned motion in the ArtiMinds RPS. This also required pulling out a drawer from a medicine cabinet. Picture taken at the demonstration days in the Hospital Karlsruhe.

Two cases must be distinguished in the real life tests: For the industrial use-case, the robotic arm had been mounted directly onto the drawer case, which means that the extrinsic calibration between robot and environment is static. Therefore no camera based object localization had to be implemented as the robot relied on a geometric environment model and the records it kept about the number of boxes in the drawers. Nevertheless the usage of a force-torque sensor in the robot’s wrist allowed to compensate for imperfections of the geometric environment model. By monitoring the measured forces, the arm motion controller was able to detect obstacles and to push medicine boxes until they aligned perfectly with the compartments in the drawers. The use of an external vacuum generator allowed the actual suction gripper on the robot to be reduced to the actual suction cup (ca. 2 cm in diameter, ca. 8 cm in length). This small size of the end effector rendered in a wide range of collision free motions in close proximity of the drawers and storage compartments. The control of the robot was realized via the RPS’s capability to generate native script code in the Universal Robot’s language. On the other hand, the real life tests in the hospital (see Figure 10) were conducted with a bulky integrated suction gripper as end effector (ca. 15 cm in diameter, ca. 12 cm in length). This reduced the freedom of movement which had to be taken into account by the RPS’s motion planning. The scenario made use of the Kinect Azure camera integrated in HoLLiE’s head, to localize the medicine boxes precisely. This was especially required whenever the robot was not perfectly aligned in front of the drawer cabinet and therefore the geometric environment model did not match reality. The distinct scenarios clarified the pros and cons of a mobile robot on the one hand and a more static scene on the other hand, also described in the upcoming section.

#### 4.5.5 Results and lessons learned

The tests on HoLLiE pinpointed the tough restrictions of the workspace due to kinematic of the PILZ arms and a missing upper body actuation (which was not used in the project). This made it impossible to open/close cabinet doors without relying on the mobility of the robot. But as combined mobile manipulation was not within the focus of this scenario, it had no negative impacts on the core tasks of stocking medicine. Nevertheless it stresses the importance of a mobile base to fulfill real-life scenarios in realistic human environments where full body motion is required as a matter of course.

The force-torque measurements in the industrial use case were facilitated to arrange the medicine boxes within the drawers by pushing them until they stacked up. This feature could not be used on HoLLiE due to the used ROS control architecture which is not compatible with the force-control of the RPS software. The setup times for the robots turned out short (less than 1 hour from unpacking the robot till first successful execution) due to the use of sensors. The automatic sensor based adaptation rendered a time intensive exact calibration unnecessary.

One important addition that was made after user feedback was the option to teach-in specific storage poses as an alternative to keying in their coordinates. The implemented ROS/RPS interaction worked out nicely so that the synergy effect of shared programming efforts between both robot systems (industrial robot and service robot) could be harnessed. Especially the facilitation of an industrial proven motion planning pipeline greatly reduced the development efforts and improved reliability.

Over all, the goal of this use case was not to prove that a robot is able to perform pick and place tasks, which is an omnipresent application of robots. Instead our prove of concept enabled non-robotics-experts to (re)program the complex task of medicine restocking individually for each hospital department and their according storage arrangements.

### 4.6 Handling of non-rigid objects

#### 4.6.1 Why this use case?

Hospital working environments stage a ubiquity of deformable and transparent objects. Handling such non-rigid objects with robots is a demanding task in the current state of the art, especially if the objects have to be grasped at a specific point. Solving those challenges adds an essential building block for robots that can handle generic manipulation tasks, and not only move rigid entities.

#### 4.6.2 Description of the use case and requirements

The example chosen to demonstrate the developed capabilities is picking a transfusion bag at the right spot, so that it can be hanged on a pole mount. This is a self-evident task for a human. On the contrary this poses significant challenges on vision-based perception systems as it requires the segmentation of the target object against the background or other objects to be able to localize it in space (position and orientation). Additionally sub-parts of the object have to be identified to retrieve the correct grasping spot for the robot. Alternatively the deformation of the object can be estimated to determine previously defined points of interest on the deformed surface of the artefact. Within the project, ArtiMinds tackled both options: Deformation estimation via energy optimization and part-aware segmentation of flexible objects via neural networks for panoptic segmentation.

#### 4.6.3 Deformation tracking via multi-step non-linear solver pipeline for energy minimization

**FIGURE 11:**
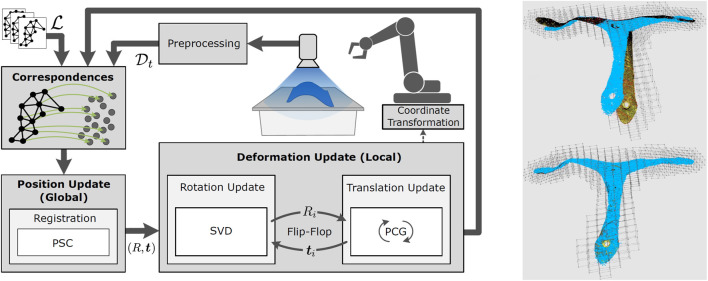
Left: Processing loop of high dimensional optimization strategy to enable grasping of deformed objects; Right: Example object with highly accurate deformation estimation and model adaptation. Source of both images: Dittus et al. (2021).

Deformable objects represent an infinite number of degrees of freedom and are therefore hard to be modeled and tracked over time. The ArtiMinds tracking approach was developed in the work of Dittus (2019). It combines several state of the art solutions for rigid object state estimation, physical modelling (such as deformation fields) and computer graphics (such as projection) into a processing pipeline to realize a marker free and deformation model free approach (see Figure 11). Furthermore, “a discretized deformation field [is used], which is estimated during runtime using a multi-step non-linear solver pipeline. The resulting highdimensional energy minimization problem describes the deviation between an offline-defined reference model and a pre-processed camera image. An additional regularization term allows for assumptions about the object’s hidden areas and increases the solver’s numerical stability”, as stated in the later publication by (Dittus et al., 2021, p. 1).

#### 4.6.4 Part-aware panoptic segmentation

**FIGURE 12:**
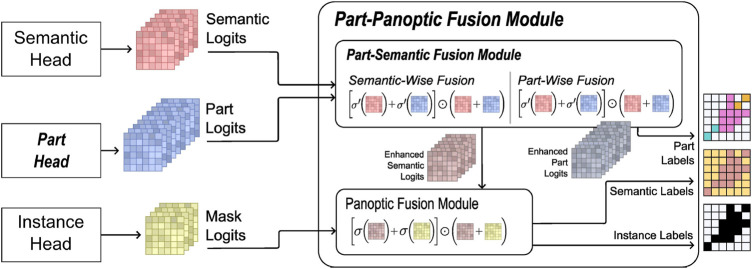
Architecture of the part-panoptic fusion module that shows subnets and processing steps which deliver labels for parts, instances and semantics in separate image channels. Source: Alt et al. (2023).

Panoptic segmentation describes neural networks originally used in autonomous driving scenarios. It delivers a robust environment segmentation while it can keep different instances of the same object classes apart. The work of Nguyen (2022) extends the EfficientNet architecture from Mohan and Valada (2021) with a so called “Part segmentation Head” and a “Part-Panoptic Fusion Module” that are capable to identify part-whole relationships and therefore essential components of objects. The network architecture can be seen in Figure 12.For the successful training of the networks, large numbers of training example images were required. Featuring different translucent objects in front of varying backgrounds and from different perspectives. To generate these vast scene variations efficiently, a robot mounted camera orbited a horizontally mounted display which showed artificial backgrounds for the object placed on the screen. Two methods for the automatic segmentation of the training data are described by Alt et al. (2023), which allow unsupervised capturing of example scenes.

#### 4.6.5 Real life tests

**FIGURE 13:**
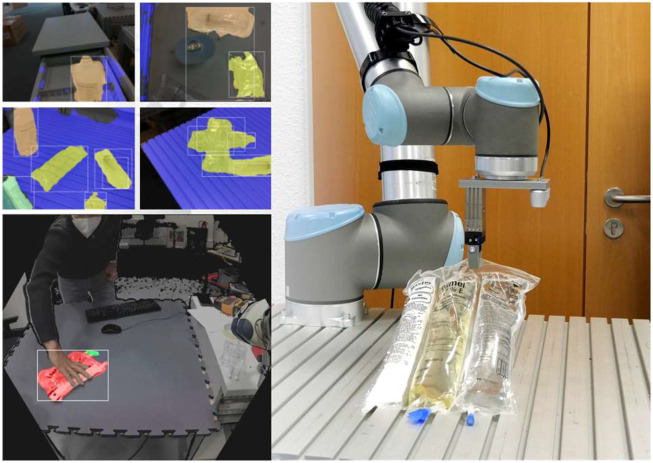
Top left: Segmentaion results of transluecent infusion bags. Bottom left: Part segmentation (bag and cap). Right: Targeted grasping of a transluecent infusion bag at the lower end center by a robot after successful part segmentation. Source of both images: Alt et al. (2023).

A full blown manipulation scenario on the HoLLiE robot could not be evaluated in a hospital scenario. This would have required the integration of additional precision gripper fingers which was not possible due to the time constraints of the project. Therefore only the perception part with a live camera stream from the Kinect Azure camera in HoLLiE’s head could be demonstrated. During the showcase, the part-aware segmentation ran natively on HoLLie’s computers. This was possible due to its encapsulation inside a ROS node. It also allowed to use the ROS rviz tools to visualize the segmented and annotated video stream for the audience. The images (comparable to the left side of Figure 13) showed the live tracking of a translucent medical infusion bag and its cap, while a person moved and deformed the item.

In contrast to the hospital scenario, a lab setup with a Universal Robots UR10e showcased the robotic manipulation side. For this, the output of the described segmentation and tracking algorithms was forwarded to the RPS’s plugin interface for the motion planning task. It derived a collision free grasping motion to the center of the bags lower end, where the hanger is located (i.e., on the opposing side of the detected cap). This pipeline enabled the UR10e robot to grasp the bag successfully at the handle from 15 different poses after at most 3 trials despite various deformations (see right side of Figure 13) of the bag.

Also for the deformation estimation based on energy optimization we did test runs in the lab to demonstrate and measure the precision that can be achieved: With three different objects (sealing rubber part, octopus camera mount, human hand) with a size of about 30 cm and deformations of several centimeters the UR10e robot was able to hit a specific point on the object surface with an positional error less than 1 mm. These results outperformed the state of the art, regarding the precision but also the algorithm’s run time. For details refer to Dittus et al. (2021).

#### 4.6.6 Lessons learned

The robotic manipulation of deformable objects is a highly relevant task in the hospital or nursing environments where such objects are part of the daily routine work. Therefore our work is not only a prove of concept but a crucial enabler for robotic systems in this field. Again, synergies with the manufacturing industries could drive further developments, as those would profit from technological advances to handle foam insulation, hoses, seals and so on. Within the project we could achieve great progress on the scientific side: We published new approaches with neural networks as well as with numerical optimizers that can be deployed in a variety of scenarios. The most important conclusion for translucent objects was, that 2D RGB images are a lot more reliable to acquire and process than 3D pointclouds. Our robotic setup that we used to gather training data in an almost unsupervised manner will be helpful for further research.

## 5 Discussion

The implementation of service-robotics in the real world bears challenges regarding the social aspects, especially in the context of care-giving. But apart of that, also the purely technical side still offers a lot of unsolved problems, regarding hard- and software of robots (Krüger and Dolriis, 2018). The current hype of AI as a solution to all robotics problems can only partly compensate hardware shortcomings and will also not solve social aspects, such as human-robot trust (Obaigbena et al., 2024), so quickly (Brooks, 2017). Nevertheless, the HoLLiECares project tackled highly ambitious goals in various service-robotics scenarios. Those scenarios were chosen to not require the dexterous skills of human hands, including tactile perception and sophisticated manipulation control. That way we could deliver strong solutions to most of the examined challenges, while pushing the envelope of robotics research. Our results clearly show, that also a technical solution which is not on the performance level of a human, can still be of significant help. It can take the load of dull and repetitive tasks off caregivers so they can focus on their valuable key competences which is a tangible benefit for patients, care attendants, and the institutions.

The proof of concept for the six selected use cases was successful. Table 2 shows a list of the investigated use cases divided into the implemented sub-functions and an assessment of the extent to which the sub-functions passed the proof of concept. As can be seen there, the use case “Pushing a wheelchair” was divided into four sub-functions: the grasp and steering of the wheelchair with a robot was successful as well as the trajectory planning for the coupled system consisting of the robot and the wheelchair. Pushing the wheelchair with a mannequin was partially successfully demonstrated, while pushing the wheelchair with a real person was not possible. The use case “Autonomous Accompaniment to Examination Rooms” consists of three sub-functions: The development and evaluation of the haptic support device for patients was successful as well as the simulative trajectory planning. The real cooperative trajectory planning was not successful. The use case “Instructions for Body Movement” had three sub-functions: Audiovisual instructions worked out successfully. Voice communication for inquiries only partly worked. The exercise detection with correction of incorrect exercises worked successfully. The use case “Wound Documentation” had two sub-functions of which the ASR Speech Recognition worked and the context-sensitive understanding of speech did partly work. The use case “Storing medicine” consists of four subfunctions, all of which were successfully demonstrated. In the use case “Handling of non-rigid objects” the deformation estimation to locate points of interest as well as the grasping and placing of non-rigid objects with a UR robot worked successfully. The segmentation of translucent objects and sub-parts only partly worked and the grasping and placing of non-rigid objects with HoLLiE did not work. Even if some of the sub-functions mentioned could not be successfully demonstrated, the respective core functionalities were at least successfully demonstrated in a simulation. Based on these developed core functionalities, it can be seen that the use cases can be implemented conceptually. This finding motivates further research in these use cases with a mobile care robot.

**TABLE 2:** List of use cases and evaluation of the proof of concept based on implemented sub-functions. Evaluation: ✓ = worked, • = partly worked, ✗ = did not work.

Use case	Sub-functions with proof of concept assessment	
Pushing a wheelchair	Grasping and steering a wheelchair with a mobile robot	✓
	Trajectory planning for coupled robot and wheelchair	✓
	Pushing and steering the wheelchair with mannequin	•
	Pushing the wheelchair with human	✗
Autonomous accompaniment to examination rooms	Haptic support device for patient on the robot	✓
	Cooperative trajectory planning (simulative)	✓
	Cooperative trajectory planning (real)	✗
body movement	Audiovisual instructions	✓
	Voice communication for inquiries	•
	Correction of incorrect exercises	✓
Wound documentation	ASR - Speech Recognition	✓
	Context-sensitive understanding	•
Storing medicine	Identification and 3D localization	✓
	(Re-) Programming for non-experts	✓
	Operating drawers, grasping and placing with HoLLiE	✓
	Operating drawers, grasping and placing with UR robot	✓
Handling of non-rigid objects	Segmentation of translucent objects and sub-parts	•
	Deformation estimation to locate point of interest	✓
	Grasping and placing with HoLLiE	✗
	Grasping and placing with UR robot	✓

As mentioned above, another finding from the project is that the modified HoLLiE robot does not provide the most suitable hardware for every investigated use case, but rather only served as a robotic test platform. During the development of the use cases, the robot’s physical limitations became apparent: safe robot arms available on the market for human-robot collaboration applications are not yet available for loads with a full human body weight. In addition, the robot’s dimensions (wide platform, wide shoulders) were actually too large for use in a nursing ward. More market-ready robots must be much more compact in this regard. At the same time, the constraints for sorting medication boxes in sometimes narrow ward rooms require a very narrow size for a robot. This could in turn have a negative impact on the necessary stability for accompanying a patient or pushing a wheelchair with a patient. This means that in future multifunctional care robot developments, either the design of the multifunctionality in the combination of use cases must be reconsidered or the spatial situation of the hospital ward must be adapted to the robot’s dimensions, such as storing medication in larger rooms that are more accessible to the robot. In addition, for a successful implementation of the use cases in the future, a new robot specializing in the investigated use cases mustbe developed.

In addition to physical limits, process limits within the use cases were also apparent, which cannot yet be fully covered by a robot. The use cases considered here always assumed an ideal use case execution. Edge cases such as a patient refusing to take part in a body movement exercise, fainting when being accompanied to an examination room, or leaving the robot could not be covered. In these cases, a human caregiver outperforms a robot through the ability to react spontaneously to unforeseen, patient-specific events. However, the use cases were deliberately considered ideal within this research project for the proof of concept of a multifunctional care robot in order to obtain an initial indication of the feasibility of such a robot. Further research must follow in order to cover all the edge cases mentioned.

Looking at the selection of use cases, it can be said that the use cases that were developed here in close cooperation with nursing staff are generally relevant for use in hospitals. Of course, they are only exemplary: the requirements in other hospitals could be different. In addition, individual use cases could be better implemented in other ways. For example, a tablet would be sufficient for wound documentation. And for medication storage, a stationary system would be less susceptible to potential disruptions. On the other hand, this question is also related to the combination with other functions. If, for example, the wound documentation use case is integrated into a robot with arms, it makes sense from the point of view of the nursing staff to add the function of transporting wound dressing materials (which usually are non-rigid objects). And a robot that can handle medicaments can be useful in a hospital. The question, which functions can and ideally should be adressed with a multifunctional robot for nursing care needs further research. Core requirements are work facilitation functions, such as transportation and logistical tasks. That was shown by our project as well as the ones listedin section 1.

In addition to the successful technical development, another aspect became relevant. It became clear that it is not sufficient to break down the work steps performed by nurses, for example, into the smallest possible and most concrete individual steps and then have them performed by robots. Such an approach would be too naive. Rather, it has been shown that the use of robots in particular and of technology in general in nursing also changes the field of work itself. In addition to activities such as transporting or transferring patients, nurses work on building relationships with patients or perform the very specific nursing task called patient observation (Brünett et al., 2024). Such activities are regularly performed besides other tasks and activities. In section 1 we showed that many of the functions of robots in nursing revolve around work facilitation for nursing staff. Professional nursing care does not just consist of several independent tasks; nursing care is much more complex than this. If robots take over specific tasks, it has to be considered, which other aspects of nursing care are influenced and possibly obstructed by that. These aspects must be given greater consideration in the development of robots and general technical solutions for nursing in the future.

## Data Availability

The raw data supporting the conclusions of this article will be made available by the authors, without undue reservation.
